# Loss of the homeostatic protein BPIFA1, leads to exacerbation of otitis media severity in the *Junbo* mouse model

**DOI:** 10.1038/s41598-018-21166-7

**Published:** 2018-02-15

**Authors:** Apoorva Mulay, Derek W. Hood, Debbie Williams, Catherine Russell, Steve D. M. Brown, Lynne Bingle, Michael Cheeseman, Colin D. Bingle

**Affiliations:** 1 Academic Unit of Respiratory Medicine, Department of Infection, Immunity and Cardiovascular Disease, University of Sheffield, Sheffield, UK; 2 MRC Mammalian Genetics Unit, MRC Harwell Institute, Didcot, UK; 3 Oral and Maxillofacial Pathology, Department of Clinical Dentistry, University of Sheffield, Sheffield, UK; 4 Roslin Institute, University of Edinburgh, Edinburgh, UK; 5 Division of Pathology, University of Edinburgh, Edinburgh, UK; 6 Florey Institute for Host Pathogen Interactions, University of Sheffield, Sheffield, UK

## Abstract

Otitis Media (OM) is characterized by epithelial abnormalities and defects in innate immunity in the middle ear (ME). Although, BPIFA1, a member of the BPI fold containing family of putative innate defence proteins is abundantly expressed by the ME epithelium and SNPs in *Bpifa1* have been associated with OM susceptibility, its role in the ME is not well characterized. We investigated the role of BPIFA1 in protection of the ME and the development of OM using murine models. Loss of *Bpifa1* did not lead to OM development. However, deletion of *Bpifa1* in *Evi1*^Jbo/+^ mice, a model of chronic OM, caused significant exacerbation of OM severity, thickening of the ME mucosa and increased collagen deposition, without a significant increase in pro-inflammatory gene expression. Our data suggests that BPIFA1 is involved in maintaining homeostasis within the ME under steady state conditions and its loss in the presence of inflammation, exacerbates epithelial remodelling leading to more severe OM.

## Introduction

Otitis Media (OM), or middle ear (ME) inflammation, is the most prevalent paediatric disease and the leading cause of paediatric surgery, antibiotic prescription and conductive hearing impairment^1^. Acute OM is primarily caused by the normally commensal bacteria non-typeable *Haemophilus influenzae* (NTHi), *Streptococcus pneumoniae* and *Moraxella catarrhalis*^2^ while chronic forms of OM show a genetic component of 45–75%^3^. The large numbers of OM susceptibility genes that have been identified emphasize the importance of innate immunity in protection against OM development^4–7^. More than 20 mouse genetic models of chronic OM have been characterised in recent years and these have proven to be a powerful tool in understanding the pathobiology of OM (For review, see 8) One such model is the N-ethyl-N-nitrosourea (ENU) mutant mouse, *Junbo*, that spontaneously develops chronic suppurative Otitis media (CSOM) under specific pathogen free (SPF) conditions, characterised by development of cellular fluid and hypoxia in the ME and inflammatory thickening of the mucoperiosteum. The causative mutation is a SNP in the gene encoding the transcription factor EVI1, also known as MECOM^8,9^. EVI1 is an inducible negative regulator of NFkB^10^ and a repressor of the TGFβ signalling pathway^11^. The heterozygous mutant *Evi1*^*Jbo/*+^ mouse has increased responsiveness to microorganisms and inflammatory stimuli^10^.

The ME is an anatomical extension of the upper respiratory tract (URT) and demonstrates similar physiological and immune mechanisms. The epithelium of the URT and its secretions play a key role in protection against environmental insults and infections. BPIFA1, a member of the bacterial permeability-increasing (BPI) fold containing family of putative innate defence proteins, is one of the most abundant secretory proteins in the URT. Its expression is restricted to the nasal passages, oropharynx, trachea and bronchi^12,13^, where it localises to a non-goblet, non-ciliated population of secretory cells^14^, minor glands and mucous cells of the sub-mucosal glands^13–15^. Secreted BPIFA1 is found in nasal lavage, saliva, sputum and bronchoalveolar lavage (BAL)^13^ and in the apical surface lining (ASL) fluid from air liquid interface cultures of differentiated tracheobronchial epithelial cells^16,17^.

Alterations of BPIFA1 levels are associated with multiple URT diseases such as cystic fibrosis^18^ and chronic rhinosinusitis^19^. Despite the lack of compelling mechanistic evidence to identify the precise biological function of BPIFA1, an increasing number of studies suggest that it plays a multifunctional host defence role in the conductive airways^16^. In line with the structural similarity to BPI^20^, BPIFA1 exhibits antimicrobial activity against a variety of respiratory pathogens and deletion of *Bpifa1* in mice is associated with increased susceptibility to pulmonary infections^21–25^. Other proposed functions of BPIFA1, include a surfactant-like activity, dispersal of bacterial biofilms, and regulation of ASL volume by modulation of epithelial sodium channel (ENaC) function^16^.

Recently, evidence of BPIFA1 expression in the ME and its involvement in OM pathogenesis has started to emerge. Knocking down *Bpifa1* expression *in vivo* using siRNA in chinchillas led to impaired mucociliary clearance^23^. BPIFA1 protein was present in the effusions of patients with chronic OM^26,27^ and *BPIFA1* was also identified as one of the top hits in a Genome Wide Association Study of genes associated with OM susceptibility^28^. We recently showed production of BPIFA1 in the murine ME, *in vivo* and *in vitro*^29^ and it has been reported that *Bpifa1*^−/−^ mice greater than 10 months-of-age show increased susceptibility to spontaneous development of chronic OM^30^. Together this data suggests that BPIFA1 may be a determinant for predisposition to OM.

In this study, we have studied the role that BPIFA1 plays in protection of the ME and its association with OM development. Our data shows that although the loss of BPIFA1 did not lead to the development of spontaneous OM in mice up to 6 months-of age, it significantly exacerbates the inflammatory phenotype in the *Junbo* model of chronic OM. Our data suggests that BPIFA1 is involved in maintaining homeostasis within the ME under steady state conditions. This is the first study to report the use of compound *Bpifa1* mutants and it underlines the importance of investigating the additive effect of multiple genetic defects in order to better understand the aetiology of multifactorial diseases such as OM.

## Results

### BPIFA1 is localised to the middle ear epithelium from birth but loss of the protein is not associated with spontaneous or bacteria induced development of OM

**Figure 1:**
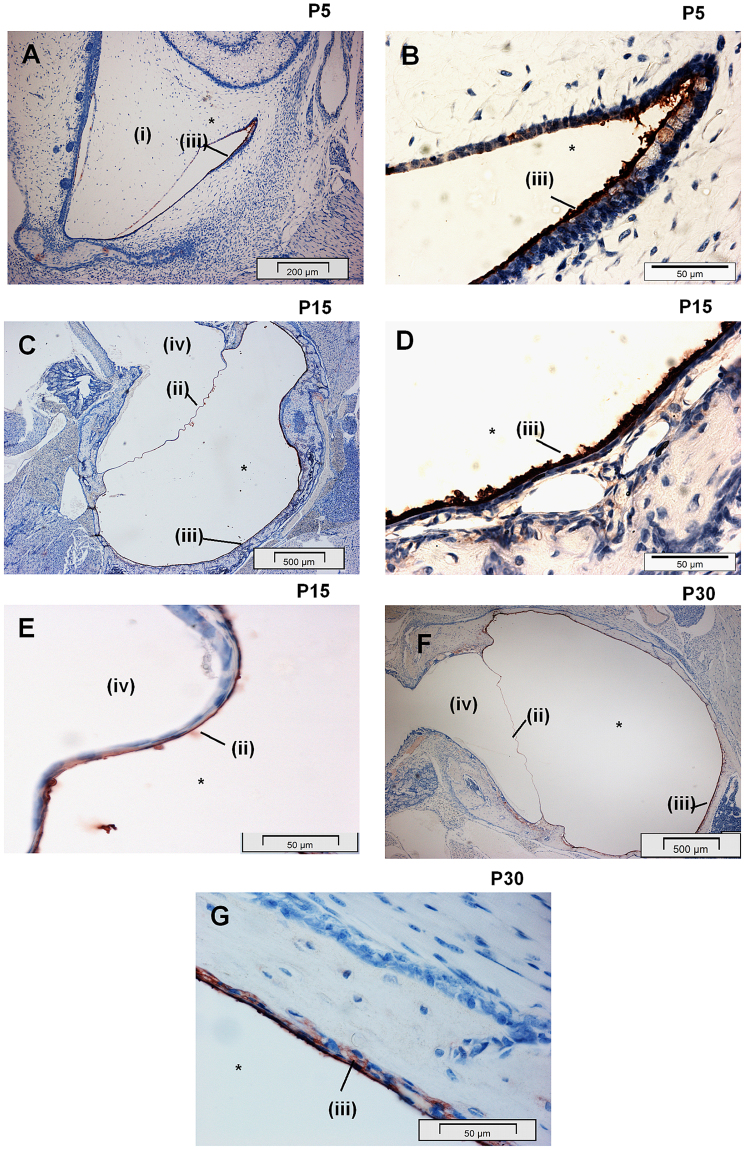
BPIFA1 is localised to the middle ear epithelium from birth. Low and high magnification images, representative of n = 3 mice showing IHC for BPIFA1 localisation in the non-ciliated epithelial cells of the developing ME cavity at P5 (**A**,**B**), P15 (**C**,**D**,**E**) and P30 (**F**,**G**). The mesenchyme which fills the post-natal ME cavity (**A**,**B**) is resorbed by P15 to form a fully air filled adult ME cavity (**C**).

We have previously reported significant production of BPIFA1 in the respiratory epithelium of the nasal passages and ME^29,31^. In order to evaluate the onset of BPIFA1 production during the post-natal development of the bulla, immunohistochemistry (IHC) was performed from the day of birth (P0) to P30 at intervals of 5 days. BPIFA1 was detected in the ME throughout this period. As described previously, between P0 to P10, the ME space is occupied by mesenchyme^32^. BPIFA1 was detected in the non-ciliated epithelium enclosing this mesenchyme mass, in the region where it retracted from the opposing epithelial surface (Fig. 1A,B). Regression of the mesenchyme began at P5, until an air filled cavity was formed by P15. BPIFA1 was localised to the non-ciliated cells throughout the ME epithelium from P15 to P30 (Fig. 1C,D,E,G). The epithelium that lines the internal surface of the tympanic membrane (Tm) also stained positively for BPIFA1 (Fig. 1F).

**Figure 2:**
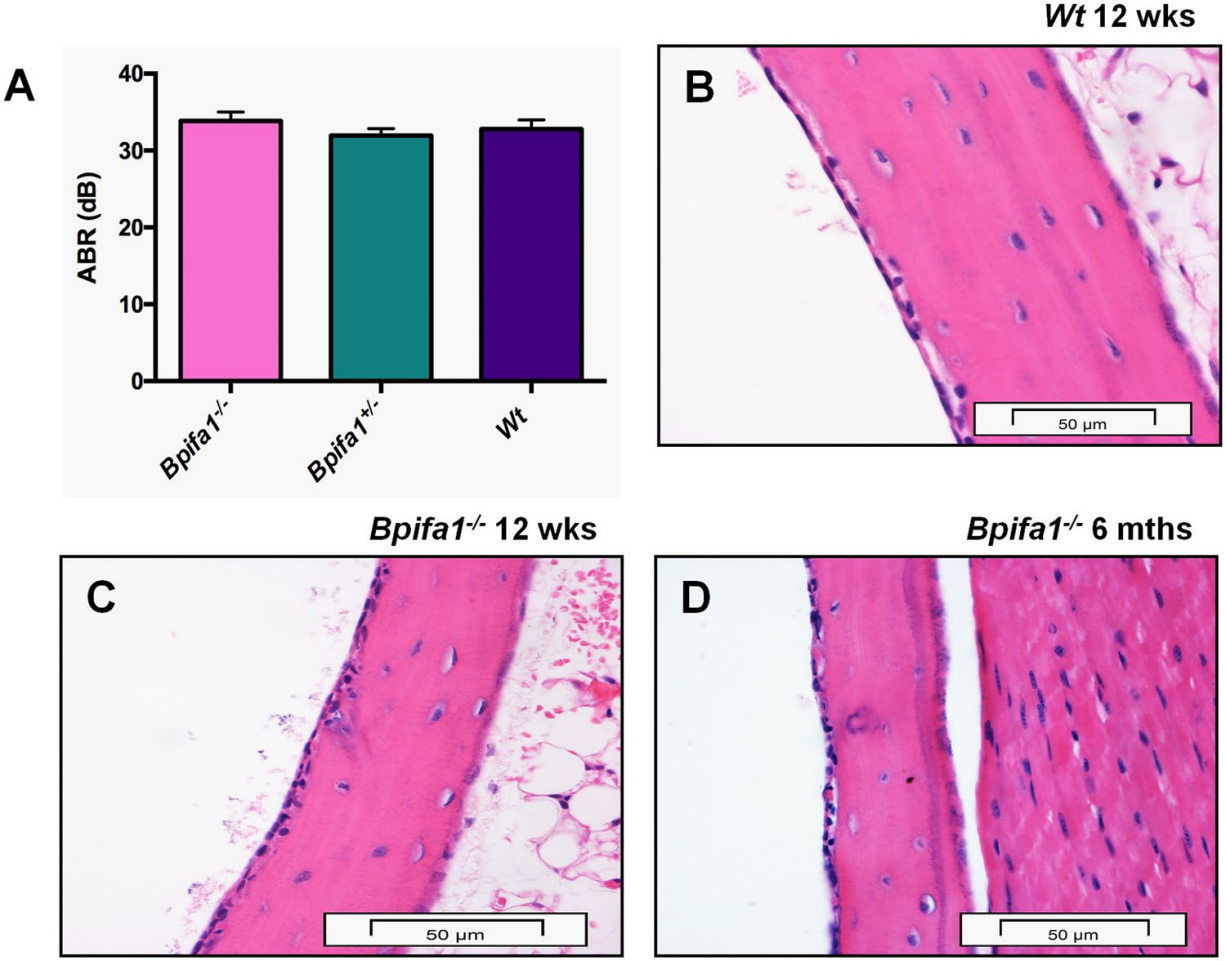
Loss of the BPIFA1 is not associated with hearing deficits or development of OM. Auditory brain stem thresholds for *Bpifa1*^−/−^ (n = 13) and *Bpifa1*^+/−^ (n = 18) mice do not differ significantly from those of WT mice (n = 9) at 12 weeks of age (**A**). p > 0.05 for One-way ANOVA with Tukey’s posthoc test. Representative High power images of H&E sections of the ME of n = 8 WT (**B**), and *Bpifa1*^−/−^ (**C**) at 12 weeks age and n = 13 *Bpifa1*^−/−^ (**D**) mice at 6 months of age show no histological abnormalities. Scale bar 50 μm for 60x objective images.

Having established that BPIFA1 is present in the ME epithelium during the process of bulla cavitation, we went on to determine whether loss of the protein caused OM. Hearing thresholds, measured using broad-spectrum Auditory Brainstem Response (ABR) (which is indicative of conductive hearing loss), did not differ significantly in *Bpifa1*^−/−^ and *Bpifa1*^+/−^ mice compared to WT mice at 8 (data not shown) or 12 weeks of age (Fig. 2A). Histological assessment of WT and *Bpifa1*^−/−^ mice did not show signs of OM development, such as middle ear mucosal thickening or fluid build-up, at 12 weeks (Fig. 2B,C) or 6 months of age (Fig. 2D) (Sup Fig. 1). No morphological differences were noted in the nasal passages, trachea or lung, (the primary sites of BPIFA1 expression) between WT and *Bpifa1*^−/−^ mice up to 6 months of age (Sup Fig. 2) and no evidence of inflammatory disease was detected following a comprehensive screen of 42 tissues of *Bpifa1*^−/−^ mice at 6 months of age (data not shown).

As the development of OM is often associated with bacterial infection, we investigated whether the loss of BPIFA1 modulated the ability of mice to respond to the intranasally (IN) inoculated otopathogen NT*Hi*. The majority of ME bullae examined after culling the mice at day 3-post inoculation, were healthy, air-filled and had intact tympanic membranes. Only a small proportion of *Bpifa1*^−/−^ (2/12) and *Bpifa1*^+*/*−^ (2/8) bullae contained fluid and 2/12 *Bpifa1*^−/−^ ears and 1/8 *Bpifa1*^+*/*−^ ears were NT*Hi* culture positive. WT bullae (n = 16 ears) did not have fluids and all were NT*Hi* culture negative. Although this data was suggestive of an effect of loss of *Bpifa1* on infection these differences did not achieve statistical significance. A similar outcome was achieved when we challenged WT, *Bpifa1*^+*/*−^ and *Bpifa1*^−/−^ mice with NTHi using baroinoculation^33,34^ and sampled bulla fluids at day 3 post-inoculation (data not shown).

Taken together, the data suggested that deletion of *Bpifa1* does not lead to spontaneous development of OM and does not lead to an increased ME infection or overt induction of OM following NTHi inoculation.

### *Evi1*^*Jbo/*+^ mice show alterations of BPIFA1

**Figure 3:**
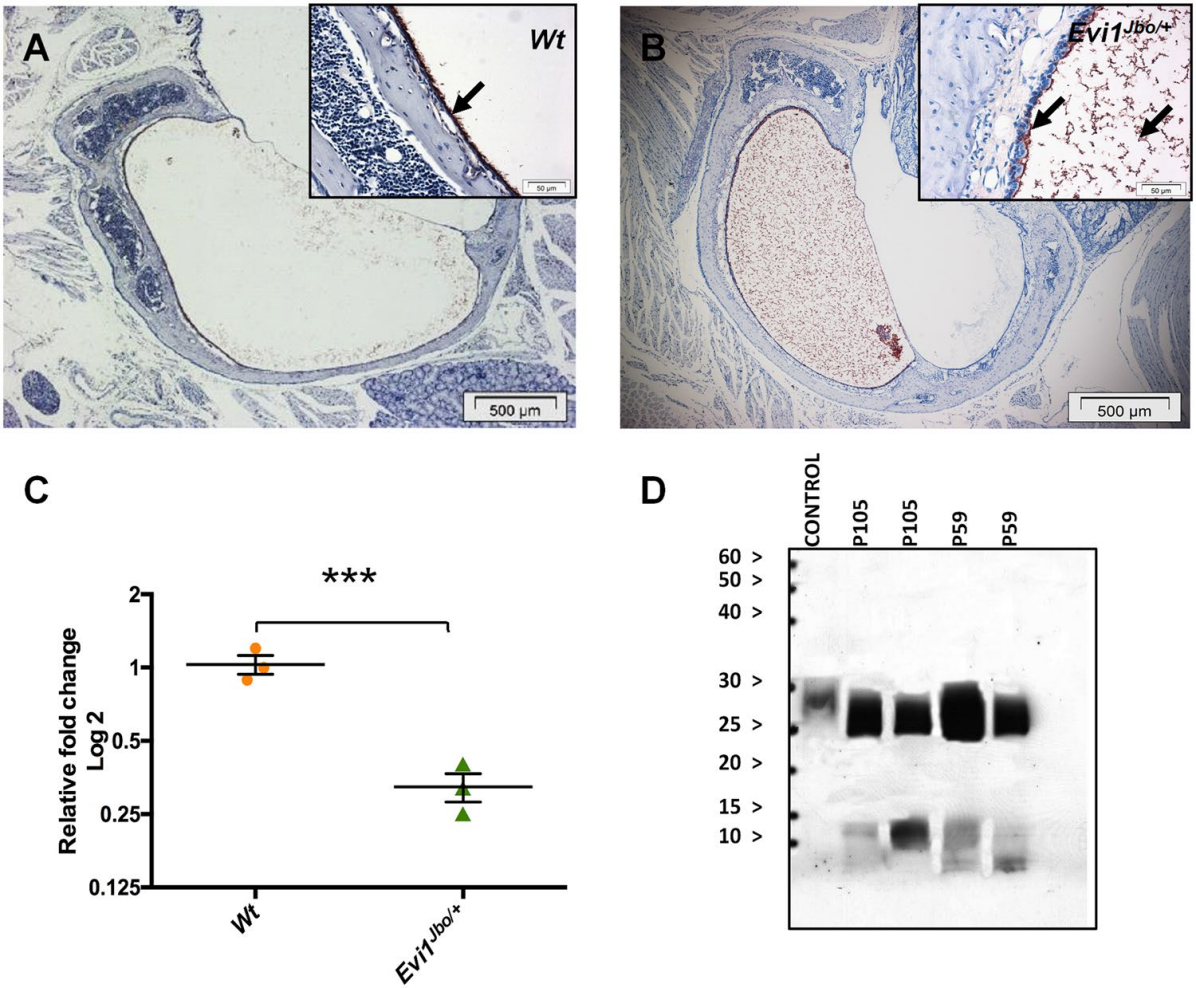
Analysis of BPIFA1 levels in the *Evi1*^*Jbo/*+^ middle ear. IHC analysis of P28 ME sections representative of n = 3 mice showing abundant staining of BPIFA1 along the WT ME epithelium (**A**) and reduction in staining intensity along *Evi1*^*Jbo/*+^ ME epithelium, but positive staining in the exudate (**B**). Arrows indicate positive staining; Scale bar: 500 μm for 4x objective images and 50 μm for 60x objective images (insets). RT-qPCR analysis of freshly isolated mouse ME mucosal epithelial cells (mMMCs) show a significant downregulation of *Bpifa1 in Evi1*^*Jbo/*+^ mucosa compared to WT mucosa. Data represented as individual RQ values ± SEM for three independent batches of freshly isolated mMMCs (each batch contains cells pooled from 6 mice). ***p < 0.001, 2 tailed t-test (**C**). BPIFA1 is readily detected in the ME effusion of *Evi1*^*Jbo/*+^ mice by Western blot (**D**). Ear exudates from two different P105 and P59 animals showed presence of the full-length protein (25 kDa) along with partially degraded peptides. The control (BAL fluid from *scgb1a1-*^*scnn1b*^ transgenic mice) showed presence of only the full-length protein.

As the loss of BPIFA1 did not result in spontaneous OM or increased susceptibility to ME infection following intranasal bacterial challenge we investigated how the protein might influence OM disease progression in an established model of OM, the *Evi1*^*Jbo/*+^ mouse. OM initiates spontaneously in *Evi1*^*Jbo/*+^ mice from P21 onwards and is characterised by significant remodelling of the middle ear epithelium^9^. At P28, IHC staining for BPIFA1 appeared to be reduced in the ME epithelium of *Evi1*^*Jbo/*+^ mice compared to their age matched WT littermate controls (Fig. 3A,B) and continued to reduce with OM progression up to P56 (Sup Fig. 3). In WT mice there was strong staining throughout the middle ear epithelium whereas staining in the *Evi1*^*Jbo/*+^ mice was seen in fewer cells and on the epithelial surface (Fig. 3B). To confirm whether the reduction in the epithelial staining intensity of BPIFA1 in *Evi1*^*Jbo/*+^ bullae was a result of a difference in epithelial gene expression, we compared expression of *Bpifa1* in freshly isolated ME mucosal cells (mMMCs) from WT and *Evi1*^*Jbo/*+^ mice using RT-qPCR. This analysis showed that there was a significant down-regulation of *Bpifa1* mRNA in *Evi1*^*Jbo/*+^ mMMCs when compared to WTs (Fig. 3C). However, BPIFA1 was clearly seen in the inflammatory ear exudates in histology sections of *Evi1*^*Jbo/*+^ mice by IHC (Fig. 3B) and by western blotting of *Evi1*^*Jbo/*+^ bulla fluid (Fig. 3D). The lack of middle ear fluid in WT mice precluded analysis of the secreted protein in these animals.

### Loss of *Bpifa1* leads to an exacerbation of OM severity in P28 *Evi1*^*Jbo/*+^ mice

**Figure 4:**
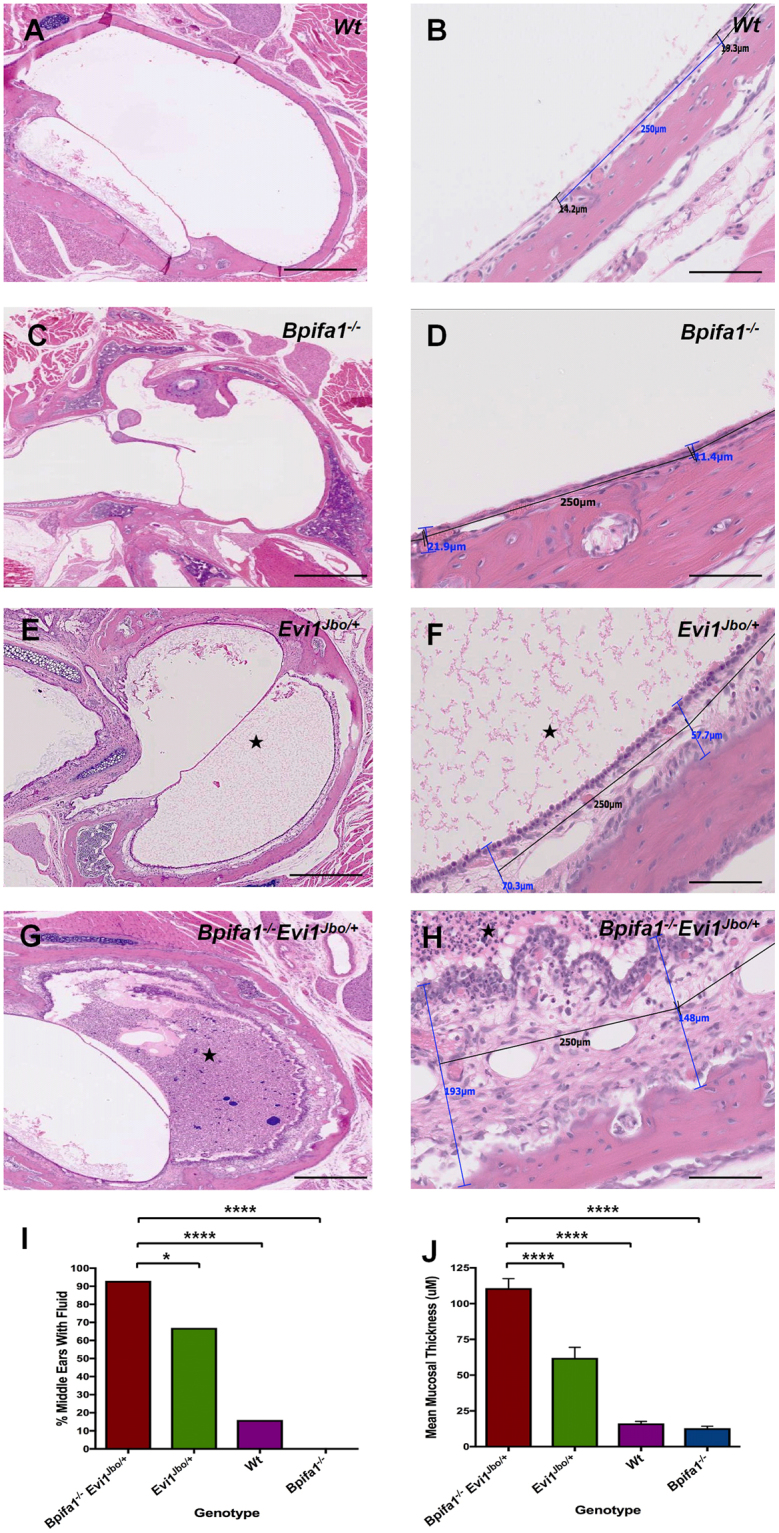
Exacerbation of otitis media severity in *Bpifa1*^+/−^*Evi1*^*Jbo/*+^ mice. Dorsal plane H&E sections from MEs of P28 WT (n = 28) (**A**,**B**) and *Bpifa1*^−/−^ (n = 12) (**C**,**D**) mice show no otitis media development. Representative H&E sections from ME of P28 *Evi1*^*Jbo/*+^ (n = 34) (**E**,**F**) and *Bpifa1*^−/−^*Evi1*^*Jbo/*+^ (n = 32) (**G,H**) mice demonstrate presence of an OM phenotype characterised by mucosal thickening and the presence of inflammatory ear fluids (marked by star). Mucosa of *Bpifa1*^−/−^*Evi1*^*Jbo/*+^ (**G**,**H**) appears thicker than *Evi1*^*Jbo/*+^ mice (**E**, **F**). Scale bar 500 μm for low magnification images and 100 μm for high magnification images. High power images show overlays used to quantitate epithelial thickness. The percentage of MEs with grossly evident fluids (Fishers exact test) (**I**) and the mean mucosal thickness (One-way ANOVA with Tukeys post hoc test) (**J**) for *Bpifa1*^−/−^*Evi1*^*Jbo/*+^ was significantly higher compared to *Evi1*^*Jbo/*+^, WT and *Bpifa1*^−/−^ mice; *p < 0.05; **p < 0.01; ****p < 0.0001.

**Figure 5:**
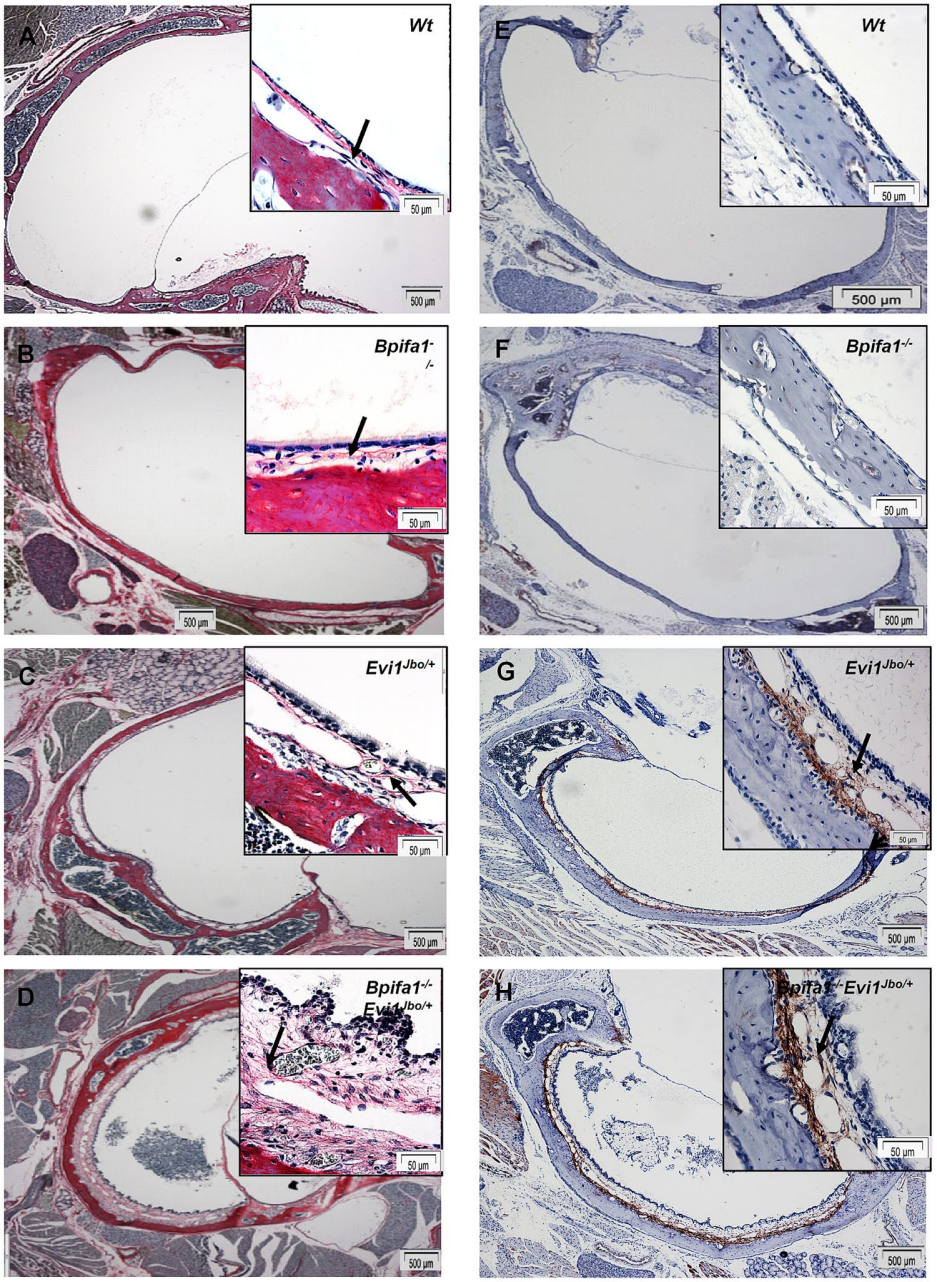
Sub-epithelial fibrosis in *Bpifa1*^−/−^*Evi1*^*Jbo/*+^ and *Evi1*^*Jbo/*+^ middle ears. Picrosirius red staining showing collagen fibres in the sub-epithelial connective tissue of WT (**A**) and *Bpifa1*^−/−^ (**B**) ME. Staining intensity increases in the inflamed and expanded mesenchyme of *Evi1*^*Jbo/*+^ (**C**) and *Bpifa1*^−/−^*Evi1*^*Jbo/*+^ (**D**) ME indicating increased deposition of collagen fibres. IHC analysis of ME sections shows no α-SMA staining in the thin sub-epithelial mesenchyme of WT (**E**) and *Bpifa1*^−/−^ (**F**) ME epithelium but intense α-SMA staining in the lower half of the expanded mesenchyme of *Evi1*^*Jbo/*+^ (**G**) and *Bpifa1*^−/−^*Evi1*^*Jbo/*+^ (**H**) ME. Arrowheads indicate positive staining. Data is representative of n = 3 mice for all genotypes. Scale bar: 500 μm for 4x objective images and 50 μm for 60x objective images (insets).

Next we tested if loss of BPIFA1 altered the prevalence or severity of OM in the *Evi1*^*Jbo/*+^ mouse. To do this we backcrossed the *Bpifa1*^−/−^ mice onto the same background as the *Evi1*^*Jbo/*+^ mice, C3H/HeH. Analysis of the ME of P28 mice showed that histological OM was highly prevalent in both *Evi1*^*Jbo/*+^ and *Bpifa1*^−/−^*Evi1*^*Jbo/*+^ mice as evidenced by microscopic thickening of ME mucosa and variable presence of bulla fluids and their cellularity (Fig. 4A–H). However, the proportion of *Bpifa1*^−/−^*Evi1*^*Jbo/*+^ ears that had grossly evident bulla fluids was significantly higher compared to *Evi1*^*Jbo/*+^ ears (Fig. 4I). Moreover, the percentage of *Bpifa1*^−/−^*Evi1*^*Jbo/*+^ with bilateral OM was significantly higher (p = 0.03) compared to *Evi1*^*Jbo/*+^ mice (Sup Fig. 4). Importantly, *Bpifa1*^−/−^*Evi1*^*Jbo/*+^ bullae showed significantly thickened mucosa compared to *Evi1*^*Jbo/*+^ ears (Fig. 3F). We looked for evidence of sub-epithelial fibrosis in inflamed *Evi1*^*Jbo/*+^ and *Bpifa1*^−/−^*Evi1*^*Jbo/*+^ MEs by staining for collagen deposition (Fig. 5A–D) and the myofibroblast marker, α-Smooth Muscle Actin (α-SMA) (Fig. 4E–H). This qualitative analysis showed enhanced staining of both markers in the *Bpifa1*^−/−^*Evi1*^*Jbo/*+^ compared to both WT and the *Bpifa1*^−/−^ mice. Thus, the data supported the observation that loss of *Bpifa1* alone does not lead to spontaneous OM development in P28 mice but significantly exacerbates the OM phenotype in *Evi1*^*Jbo/*+^ mice.

### Exacerbation of OM in *Bpifa1*^−/−^*Evi1*^*Jbo/*+^ mice is not due to an enhanced proinflammatory response, differentiation or proliferative response

As BPIFA1 has been suggested to exhibit an anti-inflammatory role we evaluated whether exacerbation of OM severity in *Bpifa1*^−/−^*Evi1*^*Jbo/*+^ mice resulted in an enhanced cytokine response. In order to test this, gene expression analysis for a selected panel of inflammatory markers was performed on RNA from freshly isolated mouse middle ear mucosal cells (mMMCs) from 7 to 10 week old *Bpifa1*^−/−^*Evi1*^*Jbo/*+^*, Evi1*^*Jbo/*+^*, Bpifa1*^−/−^ and WT mice. Although mMMCs of *Evi1*^*Jbo/*+^ and *Bpifa1*^−/−^*Evi1*^*Jbo/*+^ mice had significantly elevated levels of the pro-inflammatory cytokines *Cxcl2*, *Il1β*, *Tnfα* and the profibrotic mediator, *Tgfβ*, compared to WT mMMCs, there was no significant difference in the expression levels for any of these genes between WT and *Bpifa1*^−/−^ mMMCs or between *Evi1*^*Jbo/*+^ and *Bpifa1*^−/−^*Evi1*^*Jbo/*+^ mMMCs (Sup Fig. 3A–D), suggesting that the loss of *Bpifa1* did not modulate the cytokine response.

To evaluate if *Bpifa1* loss on the *Evi1*^*Jbo/*+^ background led to an alteration in other epithelial cell types, we investigated expression of the secretory cell marker, *Muc5ac*, and the ciliated cell marker, F*oxj1*, in *Bpifa1*^−/−^*Evi1*^*Jbo/*+^*, Evi1*^*Jbo/*+^*, Bpifa1*^−/−^ and WT mMMCs. *Foxj1* was significantly down regulated in *Evi1*^*Jbo/*+^ and *Bpifa1*^−/−^*Evi1*^*Jbo/*+^ mMMCs compared to WT mMMCs (Sup Fig. 5E). The difference in the level of expression of these markers between WT and *Bpifa1*^−/−^ mMMCs and between *Evi1*^*Jbo/*+^ and *Bpifa1*^−/−^*Evi1*^*Jbo/*+^ mice did not reach statistical significance (Sup Fig. 5E,F). This suggests that *Evi1*^*Jbo/*+^ mice show epithelial cellular alterations marked by down-regulation of *Bpifa1* and *Foxj1*, but there is no significant additive effect of *Bpifa1* deletion on expression of these epithelial cell type marker genes at this point. *Muc5ac* was significantly downregulated in *Bpifa1*^−/−^*Evi1*^*Jbo/*+^ mice compared to WT mice, but not in *Evi1*^*Jbo/*+^ mice, and there was also no significant difference between the levels of *Muc5ac* between *Bpifa1*^−/−^*Evi1*^*Jbo/*+^ and *Evi1*^*Jbo/*+^ mice (Sup Fig. 5F). Further evaluation using a larger number of mice may be necessary to unmask the potential effect of deletion of *Bpifa1* on Muc5ac levels on the *Evi1*^*Jbo/*+^ background.

In order to investigate if the OM phenotype of the hyper-proliferative mucosa in *Evi1*^*Jbo/*+^ and *Bpifa1*^−/−^*Evi1*^*Jbo/*+^ mice resulted in a cell-autonomous defect in the ME epithelium, we compared *in vitro* growth characteristics of mouse middle ear epithelial cells (mMECs) and fibroblasts isolated from the bullae of *Bpifa1*^−/−^*Evi1*^*Jbo/*+^, *Evi1*^*Jbo/*+^, *Bpifa1*^−/−^ and WT mice. No significant differences in doubling times were seen in either mMECs (Sup Fig. 5G) or fibroblasts (Sup Fig. 5H) cultured from the 4 genotypes, indicating that the cells proliferated at similar rates *in vitro*. Thus, our data suggests that there is no cell-autonomous proliferative or differentiation defect in the *Bpifa1*^−/−^*Evi1*^*Jbo/*+^ cells that explains the differences observed in the ME *in vivo*.

## Discussion

In this study we have explored the role of BPIFA1 in the context of ME host defence and OM. BPIFA1 is one of the most highly expressed proteins in the URT of humans and mice^12,13^, and forms a major component of secretions from these sites^13,17^. We have shown that the protein is present in the murine ME epithelium from birth. *Bpifa1* is expressed in the mouse nasal passages from e14.5^35,36^ whereas expression in the mouse trachea begins around the time of birth^36^. Previous reports have shown presence of BPIFA1 in the ME of mice aged 10–18 months^30^. The abundant expression of BPIFA1 in the murine ME throughout the life span suggests a homeostatic role in protection of the ME epithelium and that its loss may play a role in the progression of OM.

However, our data shows that loss of *Bpifa1* alone does not lead to the development of spontaneous OM in the mouse, up to 6 months of age. Although BPIFA1 is highly expressed in the nasal passages and trachea of WT mice, *Bpifa1*^−/−^ mice did not exhibit any overt phenotypes in these regions either. In contrast an ENU *Bpifa1*^−/−^ mutant, on a C3HeB/FeJ background, has been shown to develop low penetrance OM (30%) by 10–18 months of age^30^. This apparent difference could be due to strain differences (as we have studied mice on C57BL/6 J and C3H/HeH backgrounds) and could be further examined by studying our *Bpifa1*^−/−^ model at a comparable age. However, OM is typically studied in young mice as this better represents the timing of the paediatric disease in humans^9,28^ and the relevance of any late development of the disease is unclear. As mice over expressing BPIFA1 showed reduced levels of lung fibrosis when exposed to a sterile irritant^37^, it is possible that extended exposure to respiratory irritants or previous episodes of bacterial infection may be contributory to the low penetrance OM in aged *Bpifa1*^−/−^ mice^30^.

The loss of *bpifa1* has previously been shown to predispose mice more severe respiratory infections following an experimental challenge and yet we found that IN challenge of *Bpifa1*^−/−^ mice with the major otopathogen NT*Hi*, did not result in significant ME infection, although our data did appear to suggest that *Bpifa1*^−/−^ mice infection prevalence was marginally higher. NT*Hi* is a significant causative pathogen isolated from a majority of AOM and COME patients^38^ and the potential role that BPIFA1 plays in defence against this organism requires further studies.

Given the high levels of BPIFA1 seen in the ME, but the absence of spontaneous OM or lack of significantly increased susceptibility to ME infection by NTHi in our *Bpifa1*^−/−^ mice, we investigated the contribution of the protein to OM disease progression by studying compound mutant *Bpifa1*^−/−^*Evi1*^*Jbo/*+^ mice. *Evi1*^*Jbo/*+^ mice represent an established model of OM^9,39^ and develop hallmarks of the disease soon after middle ear cavitation occurs. Our analysis suggests that BPIFA1 protein staining was reduced in the remodelled epithelium in *Evi1*^*Jbo/*+^ mice compared to WT. It seems possible that this loss of staining may be due to an alteration of cell populations in the mucosa, with a reduction in the number of BPIFA1 producing cells being present. However we could still readily detect the protein in the ME exudates from *Evi1*^*Jbo/*+^ mice. This is consistent with proteomic data from human ME exudates^26,27^ and our own data from the apical secretions of differentiated mMECs^29^.

The most striking observation from these studies is that *Bpifa1*^−/−^*Evi1*^*Jbo/*+^ bullae demonstrated an exacerbation of OM severity compared to *Evi1*^*Jbo/*+^ mice. This suggests that removal of BPIFA1 from the middle ear environment allows for a more significant remodelling response. This further suggests a shifting of the time required to produce the OM phenotype. Indeed analysis of compound mutant mice at day P21 also revealed an exacerbation of OM severity (results not shown). It seems likely that the loss of BPIFA1 from the middle ear mucosa during the process of cavitation directly exacerbates the development of OM. Previous studies of *Evi1*^*Jbo/*+^ bulla exudates demonstrated elevation of inflammatory gene networks and hypoxia signalling genes compared to venous blood as a baseline control^39^. We have shown up-regulation of the pro-inflammatory mediators *Cxcl2*, *Tnfα*, *Il1β and Tgfβ* in the ME mucosa of *Evi1*^*Jbo/*+^ and *Bpifa1*^−/−^*Evi1*^*Jbo/*+^ mice. *Evi1*^*Jbo/*+^ and *Bpifa1*^−/−^*Evi1*^*Jbo/*+^ bullae also appeared to demonstrate epithelial cellular alterations characterised by down regulation of the ciliated cell marker, *Foxj1*, and the secretory cell marker, *Muc5ac*, and *Evi1*^*Jbo/*+^ mice demonstrated reduced levels of *Bpifa1* expression. However, despite the exacerbated OM phenotype in *Bpifa1*^−/−^*Evi1*^*Jbo/*+^ mice, there was no significant additive effect of the loss of *Bpifa1* on the expression of these inflammatory and epithelial markers. This may be a consequence of comparison based on the already inflamed *Evi1*^*Jbo/*+^ epithelium that has high basal expression levels of these inflammatory genes. At this time we are unable to identify exactly which cell type(s) express these pro-inflammatory markers but our mucosal cell isolation procedure generates a population of structural (epithelial and mesenchymal) cells without a significant inflammatory cell component.

The differences in the mucosal thickening of *Evi1*^*Jbo/*+^ and *Bpifa1*^−/−^*Evi1*^*Jbo/*+^ mice do not appear to be explained by proliferation rates of the epithelial cells and fibroblasts measured *ex-vivo*. It seems likely that inflammatory changes such as connective tissue oedema, dilation of lymphatics and blood vessels, infiltration of inflammatory cells and fibrosis are responsible for the mucosal thickening seen in the ME. An interesting feature observed in the inflamed *Evi1*^*Jbo/*+^ and *Bpifa1*^−/−^*Evi1*^*Jbo/*+^ MEs was fibrosis of the sub-epithelial connective tissue, indicated by collagen deposition and accumulation of myofibroblasts. These features are characteristic of fibrotic pulmonary diseases such as asthma and idiopathic pulmonary fibrosis^40^ and indicative of epithelial remodelling due to an over-active wound repair response following inflammation. In turn this is suggestive of over-activation of the TGFβ signalling pathway (a target for EVI1)^41^. The enhanced thickness of the *Bpifa1*^−/−^*Evi1*^*Jbo/*+^ mucosa compared to that of *Evi1*^*Jbo/*+^ mice suggests greater fibrosis due to loss of BPIFA1. BPIFA1 has been previously associated with an allergen-induced asthma-like phenotype in mice^42^ and overexpression of BPIFA1 has been shown to protect against pulmonary fibrosis^37^. A recent report suggesting a role for BPIFA1 as an epithelial derived smooth muscle relaxing factor^43^ is supportive of a potential role for this epithelial protein in mediating crosstalk with the underlying connective tissue.

An important observation in this study was the downregulation of epithelial *Bpifa1* in the inflamed microenvironment of the *Evi1*^*Jbo/*+^ ears. This reduction in BPIFA1 may represent transcriptional down regulation of *Bpifa1* or perhaps more likely is a consequence of phenotypic changes in the epithelial cell populations. Previous work with BPIFA1 has largely focused on evaluation of a role for the protein in bacterial and viral pathogen challenge studies or in the presence of pre-existing chronic allergic and inflammatory pulmonary disease (for review see^16^). Importantly, we did not observe any phenotypic abnormalities or signs of organ inflammation in the non-challenged *Bpifa1*^−/−^ mice. Thus, the presence of an infectious or inflammatory stimulus seems to be a pre-requisite to uncover functional roles of BPIFA1. This may also explain why *Bpifa1* deletion alone does not lead to the spontaneous development of OM in *Bpifa1*^−/−^ mice. Consistent with this hypothesis we have recently shown that *Bpifa1*^−/−^ mice are more susceptible to influenza A infection through a mechanism that allows *Bpifa1* deficient cells to be more readily infected by the virus^44^.

This work provides new insights into the function of BPIFA1 in the ME. From birth onwards BPIFA1 is secreted at high levels into a film that overlies the epithelium lining the ME, but its absence does not predispose to spontaneous OM or increase the susceptibility to bacterial infection. This indicates that, within normal limits, BPIFA1 is a functionally redundant innate immune protein that plays a homeostatic role in mucosal protection in a manner described for multiple respiratory proteins^45,46^. Further studies are required to address this point and whether the mechanism(s) are via alteration in surfactant function, epithelial permeability, and/or impaired antimicrobial action.

We have explored the role of BPIFA1 in mice bearing an OM predisposing Evi1 mutation, and shown an upregulation of ME epithelial cytokine expression and down-regulation of epithelial markers, with dispersal of BPIFA1 from this surface layer into bulla fluid. Deficiency of BPIFA1 on an Evi1 mutant background does not markedly alter epithelial cytokine and cell marker expression probably via perturbation of epithelial cell populations, and consequently exacerbates inflammatory thickening of the submucosal connective tissues.

Together this suggests that BPIFA1 aids homeostasis in the ME by contributing to the epithelial barrier function, and its role is revealed only under significant epithelial insult. Future time course studies are needed to define whether the deletion of *Bpifa1* on an OM prone background results in the earlier initiation of OM and whether, once initiated, the progression of disease is significantly altered. It is also important to study if loss of BPIFA1 exacerbates the OM phenotype in other OM mutant mouse lines. Given that features such as mucosal fibrosis have been noted in chronic OM in *Tlr4* and *Evi1* mutant mice^9,47^, early fibrosis, and mucosal thickening may suggest accelerated disease in the absence of BPIFA1 rather than substantially altered pathogenesis.

## Materials and Methods

### Ethics statement

Mice were bred and maintained by the Mary Lyon Centre, MRC Harwell and were housed in specific-pathogen free conditions. All animal experimentation was approved by the Animal Welfare and Ethical Review Body at MRC Harwell. The humane care and use of mice in this study was under the authority of the appropriate UK Home Office Project License.

### Mice and husbandry

*Bpifa1*^−/−^ mice on a C57BL/6 background were obtained from Professor Ralph Shohet at the University of Hawaii. The generation of these mice in which exons 2 and 3 of the *Bpifa1* gene have been deleted have been described in detail elsewhere^44^. C57BL/6 *Bpifa1*^−/−^ mice were re-derived at MRC Harwell Institute, UK and backcrossed 5 times to C3H/HeH mice to produce a congenic background. Junbo heterozygote (*Evi1*^*Jbo/*+^) mice were maintained on the C3H/HeH background^9^. Compound *Bpifa1*^−/−^*Evi1*^*Jbo/*+^ mutants and their littermate controls were generated by intercrossing *Bpifa1*^−/−^ and *Evi1*^*Jbo/*+^ mice and mice were examined at P28 and at 6 months of age. P0-P30 WT C3H/HeH mice, used for IHC of BPIFA1, were obtained from MRC Harwell Institute. For comparative purposes *Bpifa1*^−/−^ mice on the original C57BL/6 background and a mixed C3H/HeH-C57BL/6 background were used in some infection studies. Husbandry of SPF mice and microbiological surveillance is described elsewhere^9^.

### Auditory Brainstem Response (ABR) measurement

The hearing thresholds of 8 and 12 week-old mice were assessed using ABR measurements as previously described^9^.

### Tympanic membrane assessment

Mice were euthanized by intraperitoneal overdose of sodium pentobarbital (Pentoject Animalcare, UK). The mouse was decapitated and head skin removed. The tympanic membrane was examined under 10× binocular magnification to establish whether there was evidence of opacity which is indicative of macroscopic bulla fluid accumulation.

### Histology

Tissue samples were fixed in 10% formaldehyde (Surgipath Europe) for 48 hours at room temperature and the skulls decalcified in Kristenson D.F.B agent (Pioneer Scientific) for 72 hours before wax embedding. Four µm H&E stained sections of the bullae and nasal passages were scanned using the NanoZoomer Digital Pathology system and morphometric measurements made with NanoZoomer software (Hamamatsu). Thickness of the ME mucosa was measured in a defined 1 mm area in the promontory region opposite the tympanic membrane. Average mucosal thickness was calculated using 5 measurements taken at a distance of 250 μm within this region. Both male and female mice were analysed and no sex-related differences were seen in the development of OM. H&E slides for sections of a total of 42 target tissues from *Bpifa1*^−/−^ mice at 6 months of age were analysed for morphological abnormalities.

### Immunohistochemistry

Four µm wax sections were used for IHC. Endogenous peroxidase activity was blocked using 0.3% hydrogen peroxide in methanol (Fischer Scientific, UK). Non-specific binding was blocked by incubating the sections in 100% goat serum for 30 minutes at room temperature. Sections were washed and incubated in rabbit anti-Bpifa1 (1:750)^31^ or rabbit anti-αSMA (1:200) (Abcam 5694) antibodies overnight in a humified chamber at 4 °C then washed twice in PBS, incubated in 0.5% biotinylated polyclonal goat anti-rabbit secondary antibody (Vectastain^R^ Elite^R^ ABC kit, Cat No- PK-6101) for 30 minutes at room temperature, followed by incubation for 30 minutes with the ABC reagent for signal amplification before colour detection using a NovaRed system (Vector, Cat No- SK 4800). Images were captured with an Olympus BX61 light microscope and Olympus colour view digital camera.

### Preparation of NTHI bacterial inoculum and intranasal inoculation

Intranasal bacterial challenge experiments, using genomically sequenced, streptomycin resistant strain NTHi #375 (NTHi375^*SR*^), was performed as previously described^48^.

### Intranasal inoculation of mice using a barochamber

The baroinoculation protocol was adapted from previously published studies in mice^33^ and rats^34^. For baroinoculation, mice were first anaesthetised with isoflurane then intranasally inoculated with 5 μl of 1 × 10^10^ CFU/ml NTHi into each nostril and placed in a pressure chamber (Scientific workshop, MRC Harwell Institute). The pressure was raised to approximately 1.4 pounds per square inch (Psi) by compressed air through an inlet port. Thereafter, the pressure was increased by approximately 0.7 Psi every 15 secs to achieve a maximum pressure of 5.8 Psi, then after 2 minutes was decreased by venting air via an outlet port at 30 secs intervals to return the chamber to atmospheric pressure.

### Middle ear cell isolation and culture

The isolation of mMECs was performed as previously described^29^. Fibroblasts were isolated using a differential adherence method described in the same paper. Adherent fibroblasts were cultured by adding 5 ml of *mMEC basic 10% FBS media* to the tissue culture dish^29^ and incubating at 37 °C in a 5% CO2 incubator till confluent. Upon confluence, cells were washed with warm HBSS and sub-cultured twice to obtain a pure fibroblast population. For the third passage, 3mls of Trypsin-EDTA solution (Sigma) was added to the dish and incubated as above for a minute to dislodge the cells. Trypsin was neutralised by addition of 3 ml neutralising medium (*mMEC basic* plus *10% FBS*) and cells were centrifuged at 500 × g for 5 minutes. Cell pellets were resuspended in 5 mls of fresh *mMEC basic 10% FBS* and split into five T25 flasks for expansion of the fibroblast cultures. 4 ml of media was added to each flask and cells were cultured at 37 °C in 5% CO2 till confluence. When calculating population doubling time (PD), the purified fibroblasts were seeded at 5 × 10^4^ cells/well in *mMEC basic 10% FBS media* in 6 well tissue culture plates (Falcon). Similarly, fresh mMECs were seeded at 5 × 10^4^ cells/well in *mMEC- Plus media*^29^ in submerged culture. Media was changed every 48 hours. Fibroblasts were harvested every 24 hours from day1 to day 3 whereas mMECs were harvested every 48 hours by trypsinisation and quantified using a haemocytometer from day 2 to day 10.

Population doubling time was calculated using the formula.

1 $$PD=\frac{T\,\times \,Log\,2}{Log\,C2-Log\,C1}$$

where, PD = Population doubling, T = time (24 hours for fibroblasts and 48 hours for mMECs), Log = Log_10_, C1 = initial cell count, C2 = final cell count.

### Western Blotting

Bulla fluids were sampled by pipetting as previously described^48^, these were then mixed with an equal volume of 2XSDS lysis buffer. Samples were denatured at 95 °C for 5 minutes and resolved on a 12% polyacrylamide gel, transferred to a PVDF membrane using a semi dry blotting system (Biorad Trans-blot turbo) then probed with rabbit anti-Bpifa1 primary antibody (1:200^31^), overnight at 4 °C. The primary antibody was detected using a polyclonal goat anti-rabbit secondary antibody (Dako P0448) conjugated with HRP (1:2000) and bound antibody was visualised using the EZ-Chemiluminescence detection system (Geneflow, Cat No- 30500500B) and manual development. Images were scanned as a whole with no modification.

### Real time quantitative PCR (RT-qPCR)

For comparison of gene expression between WT, *Bpifa1*^−/−^, *Evi1*^*Jbo/*+^ and *Bpifa1*^−/−^*Evi1*^*Jbo*/+^ MEs, mouse mucosal epithelial cells (mMMCs), i.e. ME epithelial cells before undergoing the differential fibroblast separation, were used. Total RNA was extracted, cleaned from residual genomic DNA contamination, quantified and reverse transcribed into cDNA as previously described^29^. RT-qPCR was performed using Applied Biosystems TaqMan gene expression assays for *Cxcl2* (Mm00436450_m1), *TNFα* (Mm99999068_m1), *TGFβ* (Mm01178820_m1), *Bpifa1* (Mm00465064_m1), *FoxJ1* (Mm01267279_m1) and *Muc5ac* (Mm01276718_m1) on a 7500 Fast Real Time PCR system (Applied Biosystems), with 2× Taqman Fast Universal Master Mix (Applied Biosystems, Cat No- 4352042). 10 ng cDNA was added to each reaction and three technical replicates were performed for each assay in each batch. Target gene expression levels were normalized to three endogenous controls: *Atp5b*, *Cyc1* and *Canx* (Primerdesign geNorm^TM^ Reference Gene Selection Kit) and analysed using ABI 7500 software v2.0.1 using the 2^−*∆∆Ct*^ method. Data is presented as mean Relative quantification (RQ) and error bars represent standard error of the mean.

### Statistics

The normality of experimental data was determined by performing Levene’s test prior to the statistical analysis. GraphPad Prism (version 6.0) was used to perform all statistical tests. 2 tailed students *t*-test was used to compare two groups of data. One way or two-way analysis of variance (ANOVA) with Tukey’s post hoc test was used to compare more than two groups, for parametric data. Fisher’s exact test was used for analysing frequency data such as presence or absence of bulla fluids or genotype ratios (Chi squared analysis was used if there were more than 3 groups). Significance threshold was set at p < 0.05. Data are presented as the mean +/− standard error of the mean (SEM) unless otherwise stated. Significance levels represented on graphs are as follows: ns = not significant; *p ≤ 0.05; **p ≤ 0.01; ***p ≤ 0.001; ****p ≤ 0.0001.

## Electronic supplementary material

Supplementary Dataset 1
