# Supplementary material for: Loss of the homeostatic protein BPIFA1, leads to exacerbation of otitis media severity in the Junbo mouse model
Source: Sci Rep. 2018 Feb 15;8:3128. doi: 10.1038/s41598-018-21166-7 (PMC5814562; doi:10.1038/s41598-018-21166-7)
Supplement: Supplementary file 1 — Supplementary Dataset 1 [file 41598_2018_21166_MOESM1_ESM.docx]

**Loss of the homeostatic protein BPIFA1, leads to exacerbation of otitis media severity in the *Junbo* mouse model.**

Apoorva Mulay^a^, Derek W Hood^b^, Debbie Williams^b^, Catherine Russell^a^, Steve DM Brown^b^, Lynne Bingle^c^, Michael Cheeseman^d^, Colin D Bingle ^a^.

**Summary of Supplementary material**

Sup Fig 1 shows absence of OM in ME of *Bpifa1^-/-^* mice up to 6 months of age.

Sup Fig 2 shows absence of an inflammatory phenotype in the nasal passages and trachea of *Bpifa1^-/-^* mice which are target tissues for BPIFA1 expression, indicating that loss of the protein induced no overt phenotype

Sup Fig 3 shows that BPIFA1 expression in *Evi1^Jbo/+^* mice is high at P21 before OM develops and reduces at the onset of OM at P28.

Sup Fig 4 shows an increase in Bilateral OM in the ear of *Bpifa1^-/-^Evi1^Jbo/+^* mice compared to *Evi1^Jbo/+^* mice.

Sup Fig 5 shows that loss of BPIFA1 does not lead to a significant alteration in the levels of pro-inflammatory and epithelial gene expression above those seen in *Evi1^Jbo/+^* mice. It also indicates that the proliferative defect in ME epithelial cells and fibroblasts from *Evi1^Jbo/+^* and *Bpifa1^-/-^Evi1^Jbo/+^* mice is not cell autonomous.


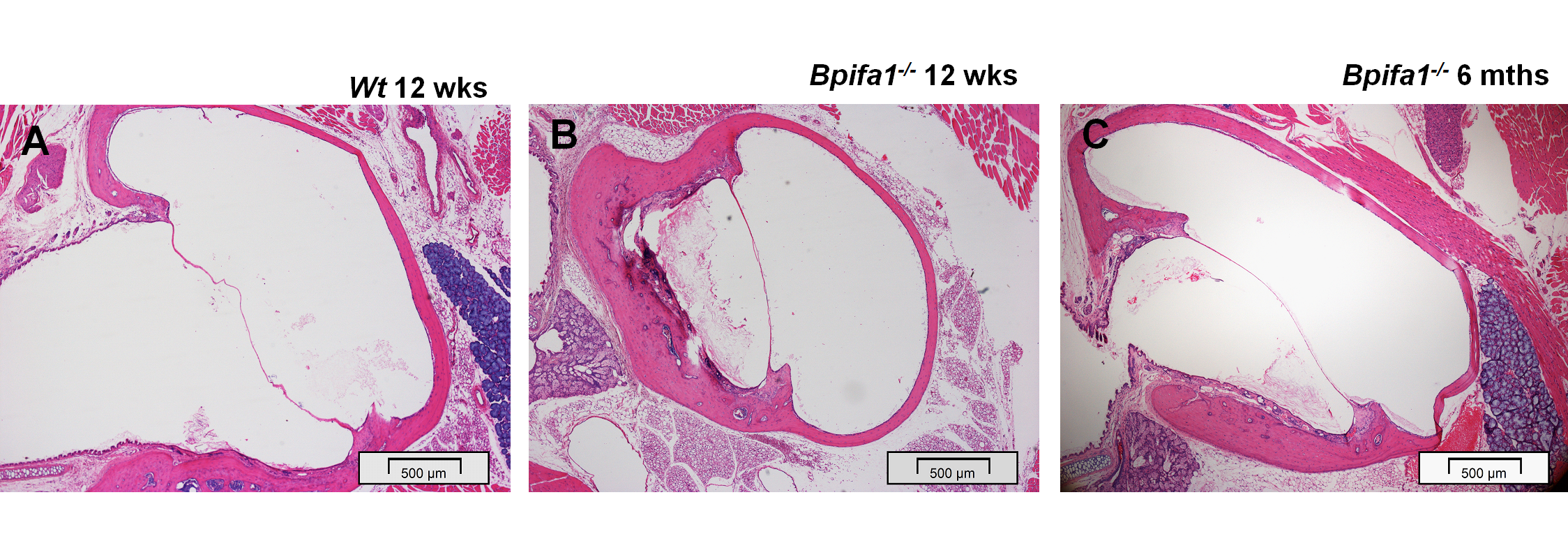


Sup Figure 1: Low power images of middle ears show that of the BPIFA1 is not associated with development of OM.

Representative low power images of H&E sections of the ME of n=8 WT (A), and *Bpifa1^-/-^* (B) mice at 12 weeks age and n=13 *Bpifa1^-/-^* (C) mice at 6 months of age show no histological abnormalities. Scale bar 500 μm for 4x objective images.

**Supplementary figures**


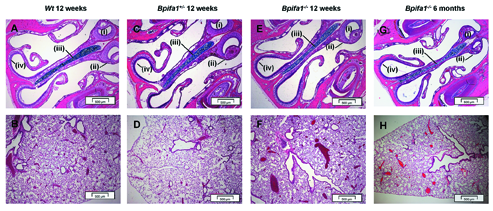


Supplementary Figure 2: Histological analyses of 12 weeks and 6 month old *Bpifa1^-/-^* mice nasal passages and lungs.

H&E sections from rostral nasal passages (A, C, E), and lungs (B. D, F) from WT*,* *Bpifa1^+/-^* and *Bpifa1^-/-^* mice respectively show no histological abnormalities at 12 weeks (n=8) of age*.* Representative H&E sections from rostral nasal passages (G) and lung (I) from n=13 *Bpifa1^-/-^* mice show no histological abnormalities at 6 months of age. Vomeronasal organ (i); respiratory epithelium (ii); nasal septum (iii) and olfactory epithelium (iv). Scale bar 500 μm.


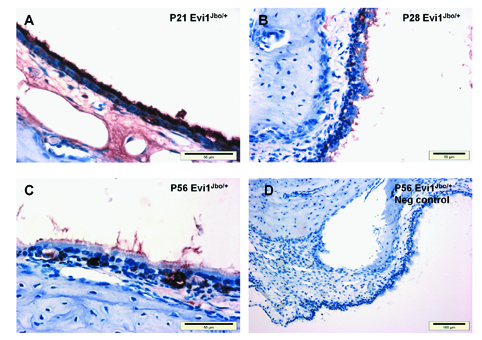


Supplementary Figure 3: BPIFA1 staining reduces with OM progression in *Evi1^Jbo/+^* mice.

IHC for BPIFA1 indicates intense epithelial staining in P21 *Evi1^Jbo/+^* mice (A) that reduces in P28 *Evi1^Jbo/+^* mice (B) and P56 *Evi1^Jbo/+^* mice (C) with OM progression. Scale bar =50 μM. Negative control omitting primary antibody (D).


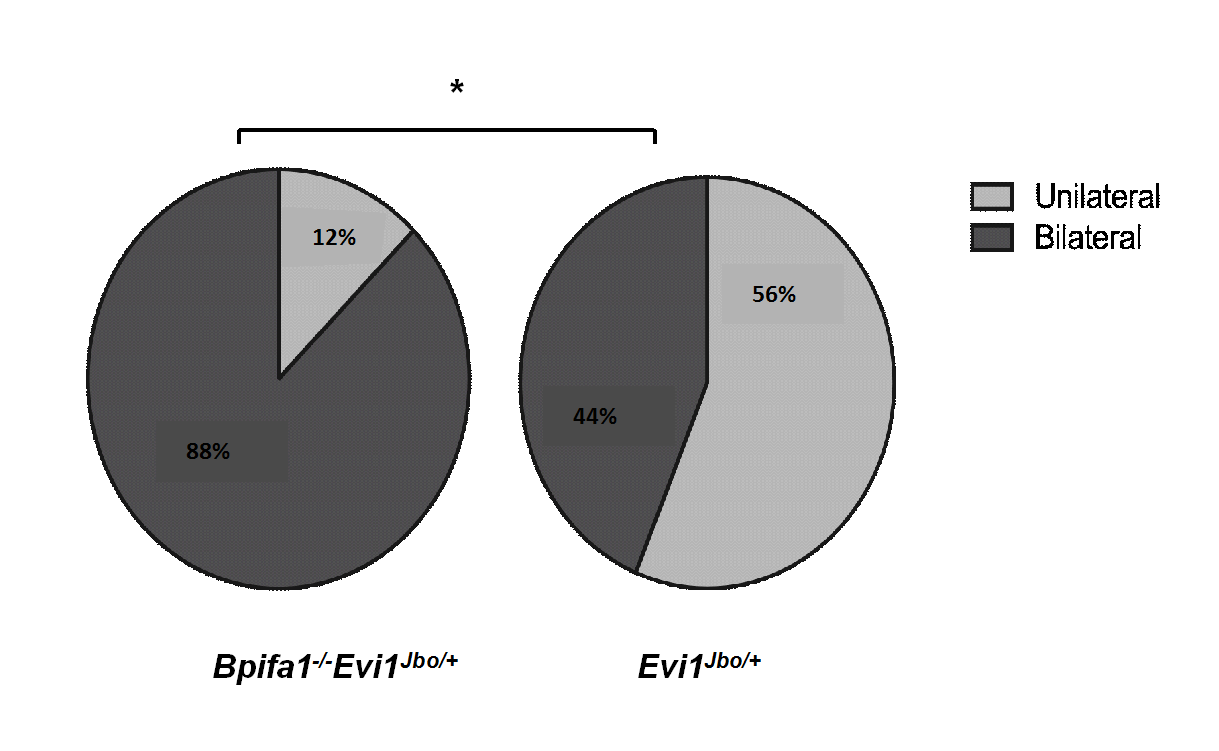


Sup Figure 4: Bilateral OM Is more prevalent in *Bpifa1^-/-^Evi1^Jbo/+^* mice.

The percentage of *Bpifa1^-/-^Evi1^Jbo/+^* mice with bilateral OM at P28 is significantly higher (*p< 0.05; Fishers exact test) than for *Evi1^Jbo/+^* mice.


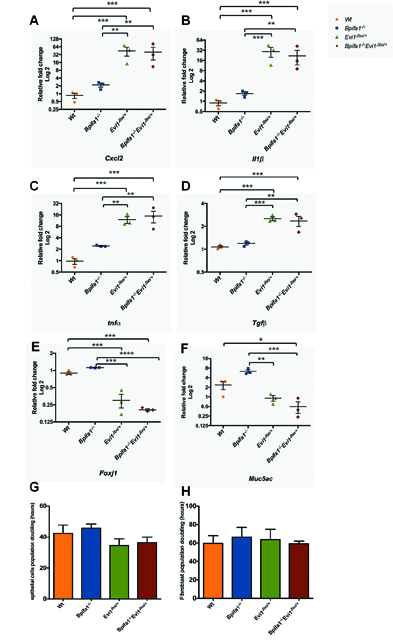


Supplementary Figure 5: Analysis of cell-autonomous defects in the middle ear epithelium of *Bpifa1^+/-^Evi1^Jbo/+^* and *Evi1^Jbo/+^* mice.

Significant up regulation of *Cxcl2* (A), *Il1β* (B), *Tnfα* (C) *Tgfβ* (D) is seen in *ex-vivo* ME mucosal cells (mMMCs) from *Bpifa1^-/-^Evi1^Jbo/+^* and *Evi1^Jbo/+^* mice compared to WT and *Bpifa1^-/-^* mice. Significant downregulation of *Foxj1 (E) in Bpifa1^-/-^Evi1^Jbo/+^* and *Evi1^Jbo/+^* mMMCs is seen when compared to WT and *Bpifa1^-/-^* mMMCs. Significant downregulation of *Muc5ac* (F) is observed in *Bpifa1^-/-^Evi1^Jbo/+^* mMMCs. Overall*,* differences in gene expression between WT *and Bpifa1^-/-^* mMMCs and between *Evi1^Jbo/+^* and *Bpifa1^-/-^Evi1^Jbo/+^* mMMCs did not reach statistical significance, indicating no additive effect of *Bpifa1* deletion on inflammatory and epithelial gene expression. There was no significant difference between the doubling times of primary mouse ME epithelial cell (mMEC) cultures (G) and cultured fibroblasts (H) between the different genotypes*.* Data were analysed using One-way ANOVA plus Tukey’s posthoc test and are represented as individual RQ values ± SEM for three independent batches (each batch includes at least 6 mice of each genotype) ****p<0.0001, ***p<0.001, **p<0.01, *p<0.05
